# Supplementary material for: Modulation of Plasma Lipidomic Profiles in Metastatic Castration-Resistant Prostate Cancer by Simvastatin
Source: Cancers (Basel). 2022 Sep 30;14(19):4792. doi: 10.3390/cancers14194792 (PMC9563053; doi:10.3390/cancers14194792)

Supplementary Information

Table of Contents

Supplementary Methods 2
Table S1 3
Table S2 5
Table S3 6
Table S4 8
Figure S1 9
Figure S2 10
Figure S3 11

Supplementary Methods

List of inclusion and exclusion criteria for the clinical trial.

Inclusion criteria

1. Males with castration-resistant metastatic prostate cancer (as per PCWG3) AND commencing docetaxel, cabazitaxel, abiraterone or enzalutamide for disease progression.
2. Age ≥ 18 yrs.
3. WHO ECOG performance status 0-2.
4. Histological confirmation of prostate cancer.
5. Adequate hepatic function with serum total bilirubin ≤ 1.5 x upper limit of normal range and ALT and AST ≤ 2.5x upper limit of normal range (or < 5.0 times ULN with documented liver metastases), serum albumin > 25 g/L, and ALP ≤ 5x upper limit of normal range.
6. Adequate renal function (with calculated creatinine clearance >50 ml/min based on the Cockcroft-Gault method, 24 hour urine or GFR scan) and serum creatinine ≤ 1.5 x upper limit of normal range.
7. Willing and able to comply with all study requirements, including treatment and biospecimen Collection.
8. Signed written informed consent.

Exclusion criteria

1. Patients already receiving a lipid lowering agent(s), or have received one in the last 4 weeks.
2. Known hypersensitivity to statins or its excipients.
3. Prior myopathy with a lipid lowering agent.
4. Active hepatic disease, including chronic active hepatitis B or hepatitis C. Testing for these is not mandatory unless clinically indicated.
5. Serious medical or psychiatric conditions that might limit the ability of the patient to comply with the protocol.

Table S1: Mean percent difference of total plasma levels of lipid classes in two comparisons.

(A) baseline lipidomic profiles with 3-lipid signature vs those without 3-lipid signature; and (B) post-simvastatin lipidomic profiles of men with 3-lipid signature vs baseline lipidomic profiles without 3-lipid signature. Statistically significant results, where p-value <0.05 by t-tests, are bolded.

**Lipid Class**	**A) Baseline lipidomic profiles with 3-lipid signature vs those without 3-lipid signature** 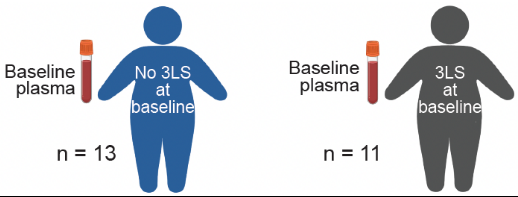		**B) Post-simvastatin lipidomic profiles of men with 3-lipid signature vs baseline lipidomic profiles without 3-lipid signature** 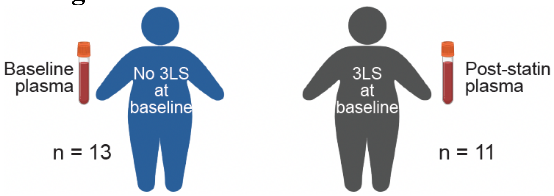	
	**Mean Percent Difference (%)**	**P-value**	**Mean Percent Difference (%)**	**P-value**
Sphingolipids				
Cer(d)	1.5	0.854	7.2	0.437
Cer(m)	-38.7	**0.002**	-12.3	0.319
C1P	28.8	0.144	19.4	0.264
dhCer	6.0	0.7	2.0	0.853
GM1	21.9	0.187	26.2	0.126
GM3	19.8	0.098	17.4	0.216
HexCer	37.6	**0.0004**	5.6	0.540
Hex2Cer	37.2	**0.01**	28.3	0.078
Hex3Cer	42.1	**0.003**	4.5	0.701
SHexCer	-3.3	0.762	16.0	0.228
SM	32.7	**0.0004**	8.7	0.225
Sph	50.2	0.072	1.5	0.899
S1P	13.3	0.408	-9.6	0.455
Glycerophospholipids				
LPC	-11.9	0.125	-9.1	0.251
LPC(O)	14.9	0.051	2.3	0.788
LPC(P)	-3.2	0.763	-13.4	0.142
LPE	-20.5	**0.037**	-8.9	0.430
LPE(P)	0.8	0.942	-5.3	0.615
LPI	-6.5	0.642	1.8	0.896
PA	-1.7	0.803	-1.6	0.839
PC	13.8	**0.036**	2.1	0.713
PC(O)	15.7	0.058	2.3	0.774
PC(P)	-0.3	0.982	-4.9	0.681
PE	-10.6	0.496	4.4	0.801
PE(O)	-17.8	0.126	-2.0	0.884
PE(P)	-16.0	0.163	-3.9	0.751
PG	-18.3	0.155	-19.8	0.195
PI	-12.2	0.078	-20.2	**0.011**
PIP1	23.5	0.173	-5.9	0.593
PS	18.3	0.606	9.9	0.659
Sterols				

CE	13.6	0.060	-5.1	0.420
COH	23.9	**0.0003**	6.7	0.137
DE	7.0	0.646	0.3	0.990
deDE	-11.2	0.620	-34.7	0.192
methyl-CE	-21.8	0.167	-3.6	0.832
dimethyl-CE	-21.7	0.150	-3.1	0.862
methyl-DE	-14.9	0.379	0.5	0.983
BA	-27.0	0.362	1.3	0.977
Fatty acyls				
AC	45.0	**0.021**	-0.6	0.971
AC-OH	36.3	**0.048**	-7.0	0.632
FFA	44.6	0.093	6.4	0.726
Glycerolipids				
DG	-7.5	0.588	-13.8	0.408
TG [NL]	-21.4	0.147	-22.8	0.196
TG(O) [NL]	-34.0	**0.010**	-6.3	0.769
TG(SIM)	-18.8	0.182	-22.3	0.177
Other				
OxSpecies	8.0	0.644	-2.3	0.839
Ubiquinone	-11.2	0.347	-14.5	0.342

Table S2: Comparison of baseline characteristics and standard treatment received with simvastatin between men who lost the 3LS and those who retained the 3LS.

T-test was performed on continuous variables. Fisher's exact test was performed on categorical variables. "n/a" = not applicable as numbers were insufficient or not different.

Characteristic	Mean (min, max), or number of men		P-value	
	Men who lost the signature (n=5)	Men who retained the signature (n=6)	t-test	Fisher's exact test
Age (years)	73 (70, 76)	76 (63, 90)	0.596	
ECOG performance:				
0-1	5	5		1.000
≥2	0	1		
Gleason grade:				
<8	3	2		0.318
≥8	0	3		
Unknown	2	1		
Site of metastasis:				
Lymph node	2	3		1.000
Bone	5	6		
Visceral	0	1		
Prostate-specific antigen (ng/mL)	92 (54, 179)	59 (0.02, 197)	0.406	
Alkaline phosphatase (U/L)	192 (64, 509)	176 (74, 564)	0.893	
Lactate dehydrogenase (U/L)	246 (174, 290)	329 (211, 518)	0.175	
Haemoglobin (g/L)	120 (94,131)	122 (102, 138)	0.824	
Metabolic risk factors:				
Diabetes*	0	0		n/a
Hyperlipidaemia	1	0		n/a
Hypertension	2	1		n/a
BMI	31 (26, 36)	25 (19, 30)	0.02	
Waist circumference (cm)	115 (104, 132)	94 (76, 109)	0.02	
Treatment:				
Taxane (docetaxel, cabazitaxel)	3 dcx, 1 cbx	4 dcx		1.000
Anti-androgen (enzalutamide, abiraterone)	1 enza	2 abi		

* There was only diabetic patient in the cohort, and his post-simvastatin plasma was not available.

Table S3: Differences in total plasma levels of lipid classes between post-simvastatin and baseline samples in men who lost or retained the 3LS.

(A) men who lost the 3-lipid signature with treatment, and (B) men who retained the 3-lipid signature with treatment. Statistically significant results, where p-value <0.05 by paired sample t-tests, are bolded.

**Lipid Class**	**A) Men who lost 3-lipid signature: Post-simvastatin vs baseline**		**B) Men who retained 3-lipid signature: Post-simvastatin vs baseline**	
	**Mean Percent Difference (%)**	**P-value**	**Mean Percent Difference (%)**	**P-value**
Sphingolipids				
Cer(d)	10.6	0.291	1.54	0.668
Cer(m)	71.8	0.081	23.03	**0.012**
C1P	-10.4	0.531	-4.54	0.803
dhCer	13.5	0.423	-16.20	0.466
GM1	-26.5	0.389	37.65	0.104
GM3	-20.0	0.079	15.99	0.066
HexCer	-35.2	**0.025**	-11.73	0.235
Hex2Cer	-22.6	0.183	9.58	0.620
Hex3Cer	-37.1	**0.026**	-16.26	0.096
SHexCer	20.8	0.112	19.17	0.193
SM	-27.2	**0.029**	-9.50	0.177
Sph	-51.6	0.234	-10.75	0.649
S1P	-45.3	0.153	9.13	0.589
Glycerophospholipids				
LPC	24.7	**0.046**	-11.79	0.336
LPC(O)	-16.5	0.076	-6.24	0.691
LPC(P)	-1.8	0.858	-17.30	0.333
LPE	42.3	**0.024**	-4.34	0.806
LPE(P)	-8.8	0.558	-3.81	0.518
LPI	8.1	0.703	9.51	0.461
PA	0.0	0.997	0.07	0.989
PC	-18.7	0.099	-2.67	0.600
PC(O)	-24.9	**0.034**	1.33	0.878
PC(P)	-12.9	0.242	2.81	0.689
PE	9.7	0.666	23.03	0.154
PE(O)	28.4	0.089	11.88	0.350
PE(P)	17.3	0.113	12.12	0.183
PG	-0.8	0.969	-2.67	0.841
PI	-4.0	0.554	-13.13	0.230
PIP1	-38.8	0.139	-8.55	0.403
PS	-35.4	0.406	25.88	**0.002**
Sterols				
CE	-16.4	0.072	-16.38	**0.001**
COH	-19.6	**0.003**	-8.62	0.181
CE+COH	-17.0	**0.04**	-15.4	**0.005**
DE	-25.7	0.147	13.84	0.765
deDE	2.7	0.963	-44.25	0.328
methyl-CE	43.2	0.156	8.75	0.536
dimethyl-CE	49.0	**0.023**	6.00	0.729

methyl-DE	48.0	0.158	-2.19	0.901
BA	26.1	0.690	50.21	0.296
Fatty acyls				
AC	-48.5	0.064	-13.01	0.666
AC-OH	-40.1	0.103	-24.06	0.371
FFA	-37.1	0.120	-16.20	0.604
Glycerolipids				
DG	-5.9	0.717	-7.73	0.635
TG [NL]	8.4	0.685	-9.50	0.549
TG(O) [NL]	111.4	0.090	2.03	0.715
TG(SIM)	3.3	0.836	-10.37	0.533
Other				
OxSpecies	27.3	0.227	-31.98	0.175
Ubiquinone	16.6	0.474	-17.81	0.073

Table S4: Differences in the plasma levels of 18 prognostic lipids between post-simvastatin and baseline samples in men who lost or retained the 3LS.

(A) men who lost the 3-lipid signature with treatment, and (B) men who keep the 3-lipid signature with treatment. Statistically significant results, where p-value <0.05 by paired sample t-tests, are bolded.

	**Lipid**	**A) Men who lost 3-lipid signature: Post-simvastatin vs baseline**		**B) Men who retained 3-lipid signature: Post-simvastatin vs baseline**	
		**Mean Percent Difference (%)**	**P-value**	**Mean Percent Difference (%)**	**P-value**
	Cer(d18:1/16:0)	-28.7	**0.038**	1.3	0.892
	Cer(d18:1/18:0)	-44.9	**0.031**	18.2	0.172
	Cer(d18:1/20:0)	-28.7	**0.010**	11.8	0.142
	Cer(d18:1/24:1)	-26.8	**0.045**	8.2	0.226
	GM3(d18:1/16:0)	-37.1	**0.024**	8.9	0.437
	GM3(d18:1/20:0)	-21.0	0.136	25.9	**0.022**
	HexCer(d18:1/16:0)	-39.0	0.051	-10.9	0.352
	SM(d18:1/16:0)	-31.5	**0.021**	-10.2	0.159
	SM(d18:2/16:0)	-23.3	0.051	-10.6	0.177
	SM(d18:2/18:0)	-32.9	0.058	-6.2	0.571
	SM(d18:2/20:0)	-18.4	0.071	-8.3	0.099
	PC(16:0/16:0)	-32.0	0.062	-6.4	0.384
	PC(38:2)	-9.4	0.501	-15.7	0.113
	PC(P-16:0/20:4)	-11.1	0.373	13.0	0.158
	COH	-19.6	**0.003**	-8.6	0.181
	PC(38:6) (a)	4.3	0.867	16.6	0.241
	PC(14:0/20:4)	97.5	**0.013**	27.5	0.403
	PC(40:8)	29.0	**0.034**	11.7	0.502

Higher plasma levels associated with poorer prognosis in previous mCRPC cohort (Lin et al 2017)
Lower plasma levels associated with poorer prognosis in previous mCRPC cohort (Lin et al 2017)

Figure S1: Baseline and post-simvastatin status of clinical lipids and 3-lipid signature in each patient.

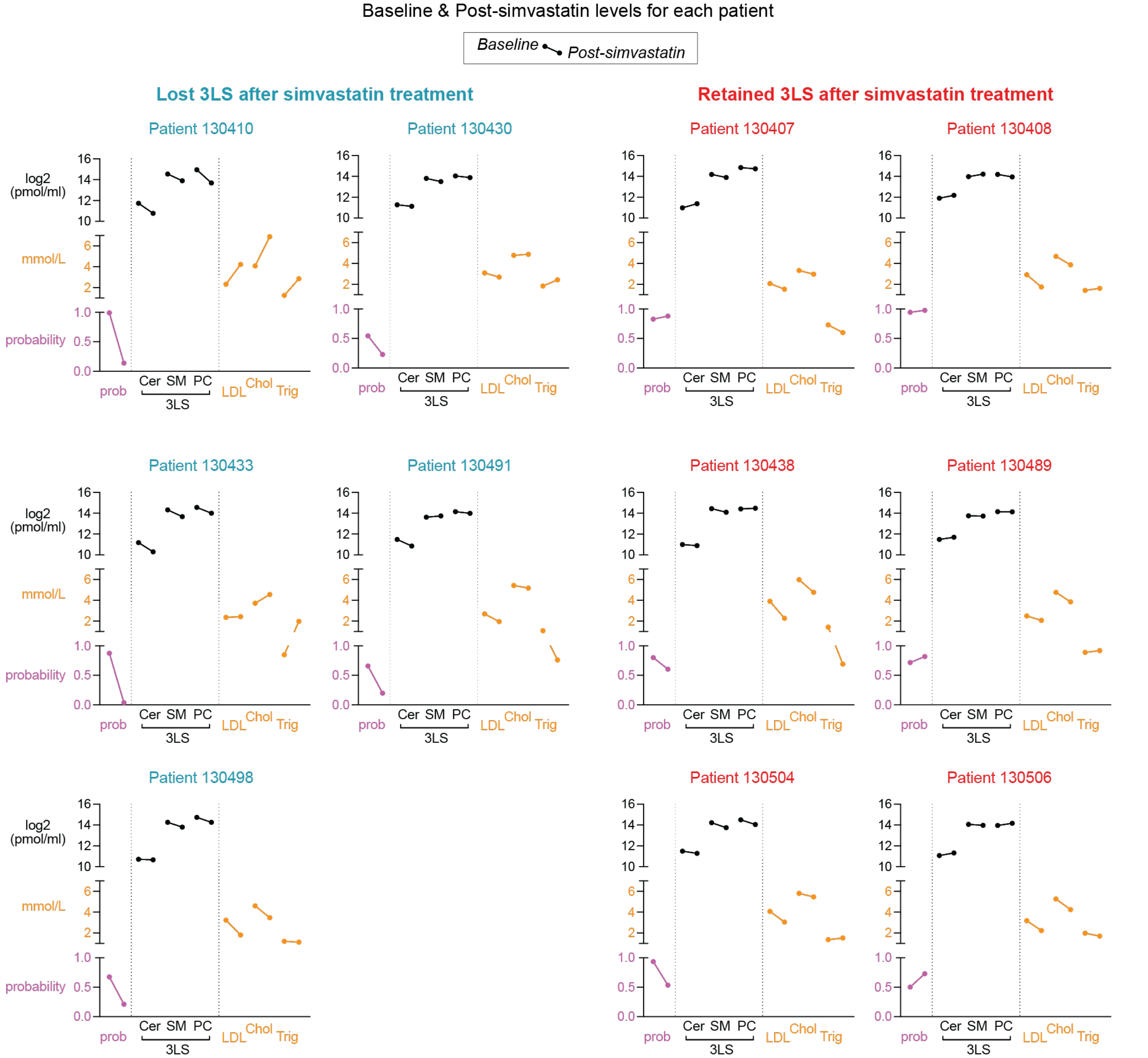

Abbreviations

prob, probability of having the 3LS (calculated from logistic regression model, see methods)
Cer, Cer(d18:1/24:1)
SM, SM(d18:2/16:1)
PC, PC(16:0/16:0)
LDL, LDL-C
Chol, total cholesterol
Trig, triglycerides
3LS, 3-lipid signature

Figure S2. Baseline and post-simvastatin status of clinical lipids and the 3-lipid signature in men who do not have the 3-lipid signature at baseline.

Four of these men gained the 3-lipid signature after simvastatin treatment.

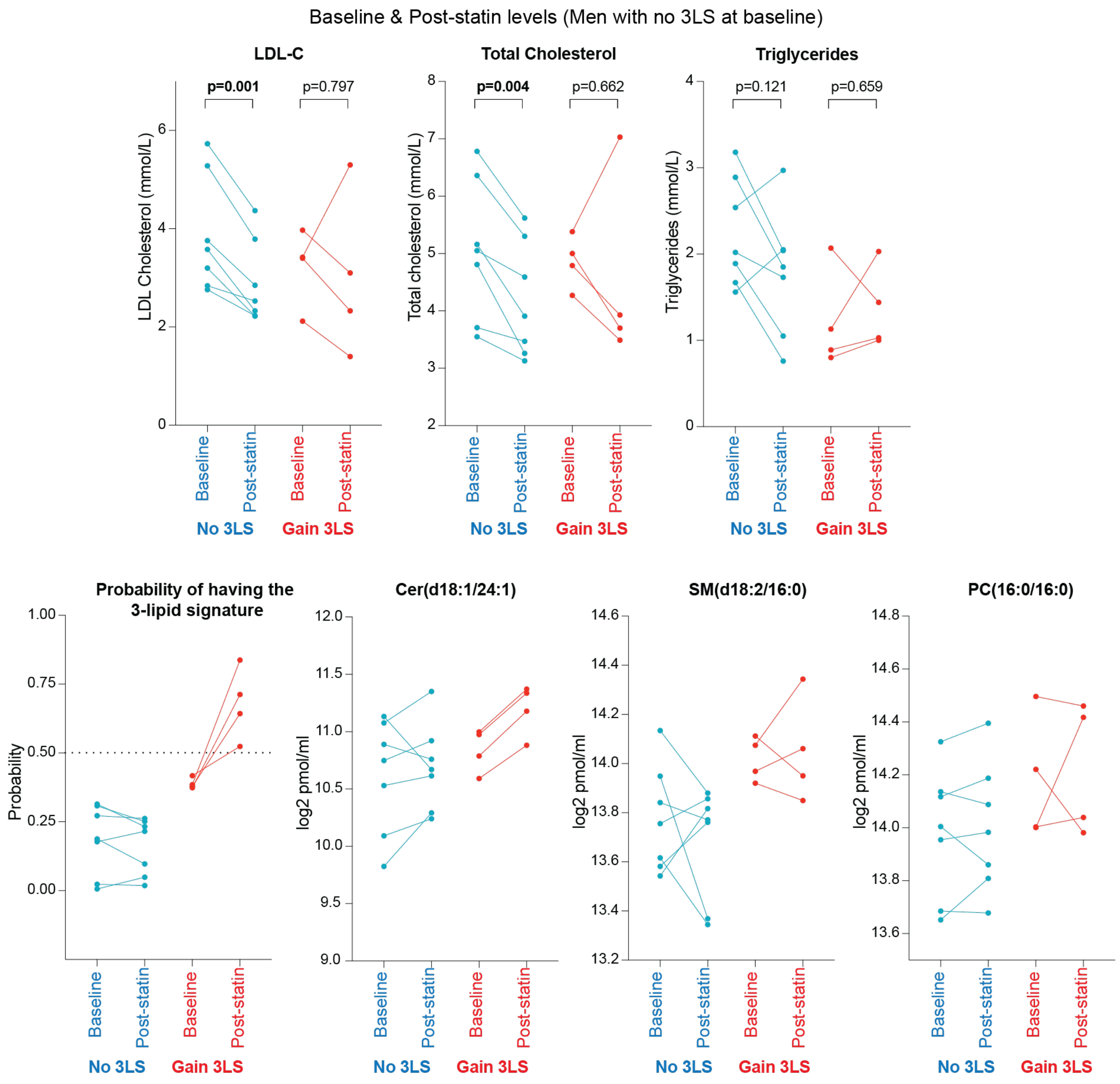

Figure S3. Mean percent difference of plasma levels of individual lipid species in post-simvastatin profiles versus baseline of men who do not have the 3-lipid signature at baseline.

(A) men who do not have the 3LS at both baseline & post-statin, and (B) men who gained the 3LS after statin treatment.

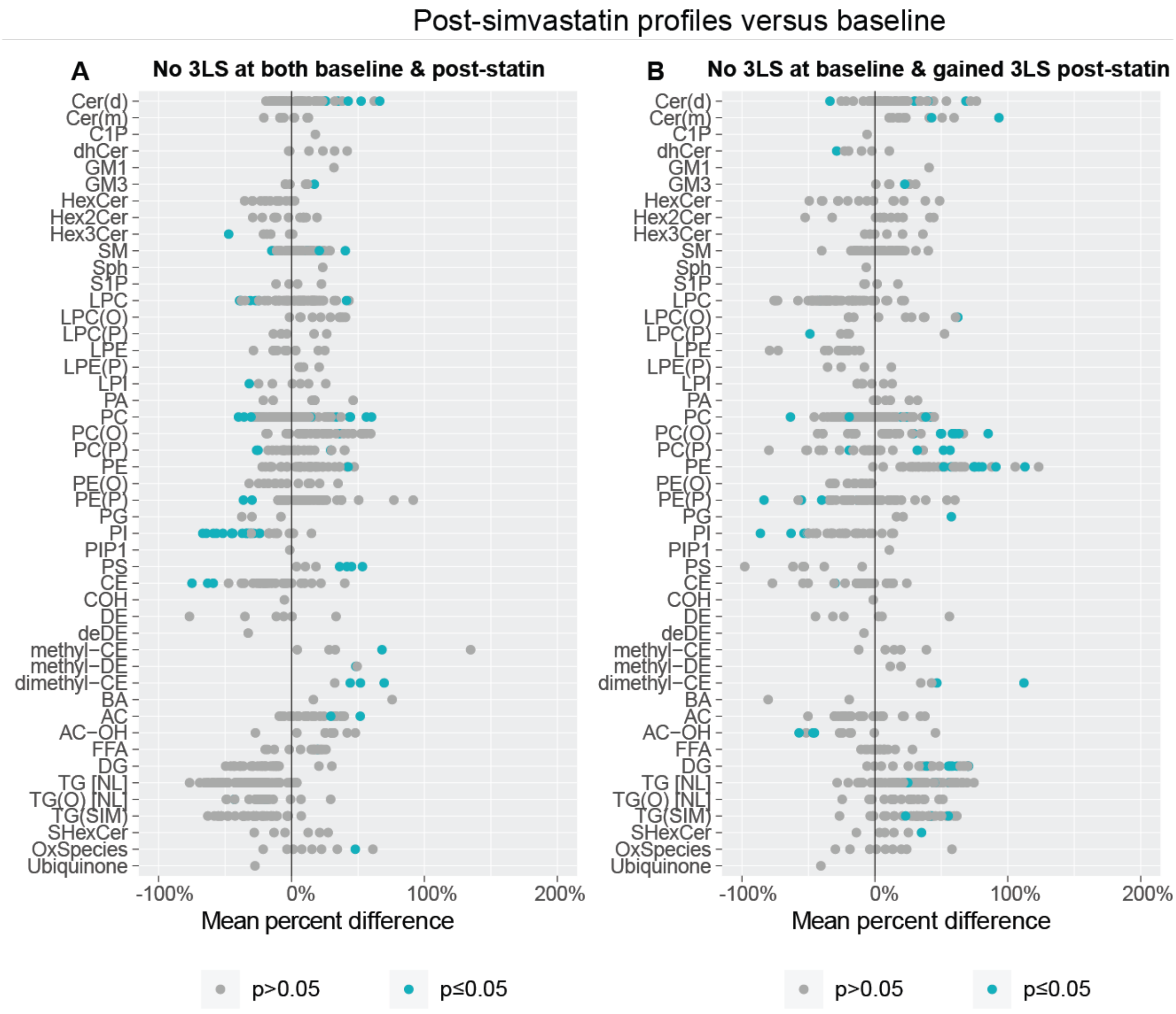

Supplement: Supplementary file 1 [file cancers-14-04792-s001.zip › cancers-1903478-supplementary.pdf]
